# Comparative Study of Gravimetric Humidity Sensor Platforms Based on CMUT and QCM

**DOI:** 10.3390/mi13101651

**Published:** 2022-09-30

**Authors:** Zhou Zheng, Guodong Zhang, Xiaomin Wang, Xu Kong

**Affiliations:** College of Electrical Engineering and Automation, Shandong University of Science and Technology, Qingdao 266590, China

**Keywords:** humidity sensing, MEMS sensor, CMUT, QCM, gravimetric sensor

## Abstract

Humidity sensors with comprehensive performance are of great interest for industrial and environmental applications. Most sensors, however, have to compromise on at least one characteristic such as sensitivity, response speed, and linearity. This paper reports a gravimetric humidity sensor based on a capacitive micromachined ultrasonic transducer (CMUT) with exceptional all-around performance, and presents a side-by-side comparative investigation of two types of gravimetric humidity sensors for a better understanding of their characteristics and sensing mechanisms. For these purposes, a circular CMUT and a quartz crystal microbalance (QCM) with a resonance frequency of 10 MHz were designed and fabricated. Poly(vinyl alcohol) (PVA) was employed as the humidity sensing layer for its hydrophilicity and ease of film formation. The electrical properties of the sensors, including the electrical input impedances and quality factors, were characterized by a vector network analyzer. The relative humidity (RH) sensing performance of the sensors was evaluated and compared from RH levels of 11% to 97%. Both sensors exhibited good repeatability and low hysteresis. The unique microscale resonant structure of the CMUT humidity sensor contributed to a high sensitivity of 2.01 kHz/%RH, short response and recovery times of 8 s and 3 s, respectively, and excellent linearity (*R*^2^ = 0.973), which were far superior to their QCM counterparts. The underlying mechanism was revealed and discussed.

## 1. Introduction

Humidity detection plays a crucial role in various applications, including environmental control and industrial processing [1,2]. For example, variations in atmospheric humidity during the fabrication of high-precision integrated circuits may require adjustment of recipes for some processes such as photolithography [3]; excess humidity may destroy some materials or change their properties during new material development [4]; and minute changes in humidity can affect biological research results [5]. Therefore, it is essential to monitor and detect humidity with precise and provident sensors.

Gravimetric sensors are widely adopted for humidity sensing due to their high sensitivity and fast response. They work by converting the mass change of water molecules into the shift of the resonance frequency. The quartz crystal microbalance (QCM) is the most popular of the gravimetric sensor types for its maturity, low cost, and capability of detecting mass changes down to the nanogram level [6,7]. The advancement of QCM-based humidity sensing techniques encompasses efforts in the enhancement of several aspects such as structure designs [8,9,10] and functional materials [11,12,13].

In recent years, an attractive candidate for humidity sensor platforms, the capacitive micromachined ultrasonic transducer (CMUT), has emerged to compete with QCMs. As another type of gravimetric sensor, CMUT is developed from micro-electro-mechanical system (MEMS) technology [14]. Hence, it possesses the general benefits that MEMS technology offers, such as easy fabrication of miniaturized arrays for selective sensing, and the capability of batch production and integration with interface circuits [15,16,17,18,19]. Moreover, it has been proven to be highly sensitive due to its micro-scale or nano-scale ultra-thin resonant structure. Lee et al. demonstrated the high sensitivity of CMUT-based humidity sensors that used 500 nm-thick single crystal silicon as the resonant membrane [20]. The relative humidity (RH) sensitivity of 2.6 kHz/%RH overwhelmed that of all QCM-based humidity sensors, which is often at the scale of Hz/%RH. Later, the sensitivity of the CMUT-based humidity sensors was further enhanced by optimizing the fabrication techniques [21] and by employing nanomaterials as the sensing layer [22]. However, these devices mostly have nonlinearity and high hysteresis.

In most situations, a more competitive humidity sensor is the one that has comprehensive performance of sensitivity, repeatability, linearity, response speed, and hysteresis. This requires the sensor and the functional material to work synergistically. Poly(vinyl alcohol) (PVA) is a low-cost, non-toxic, and biodegradable polymer with outstanding mechanical and film-forming properties, chemical and thermal stability, and water-solubility [23]. These features have contributed to diverse products such as surgical threads, resins, and lacquers [24], and make PVA and its composites excellent materials for sensing applications [25,26,27]. The ease of film formation is helpful for surface modification of the CMUT, and the abundance of hydroxyl groups is favorable for a high relative humidity (RH) sensitivity. Therefore, we have been motivated to develop a CMUT-based humidity sensor using PVA as the active layer.

As an increasing number of chemical sensors are emerging, comparative studies are necessary for a better understanding of their characteristics and sensing mechanisms. Robinson et al. compared capacitive- and resistive-based transduction mechanisms using single-walled carbon nanotubes [28], and concluded that the capacitive detection method was more sensitive and reliable. Zeinali et al. presented a comparison between interdigitated electrode- (IDE-) and parallel plate-based capacitive sensors using metal-organic frameworks as the sensing layer for the detection of volatile organic compounds (VOCs) [29]. The IDE structure was reported to have superior recovery speed and repeatability, but inferior sensitivity and low limit of detection. Chappanda et al. compared QCM- and IDE-based acetone sensors using zeolitic–imidazolate framework as the sensing material [30]. The IDE capacitive sensor results in higher sensitivity and smaller device size, but poorer selectivity to acetone with respect to humidity and poorer thermostability. Although the advantages of CMUTs over QCMs were have been previously mentioned in general in limited reports, the comparisons are insufficient in terms of their sensing characteristics and the underlying mechanism. A detailed comparative study is in strongly in demand.

In this paper, we developed a high-performance CMUT-based humidity sensor and presented a side-by-side comparison with a QCM counterpart to provide more insights into the two types of gravimetric sensors. The CMUT and QCM humidity sensors based on the PVA active layer were designed, fabricated, and characterized. The RH sensing properties of the two sensors, including the sensitivity, linearity, reproducibility, repeatability, response speed, and long-term stability were experimentally studied and compared. The underlying mechanism that caused the distinct sensing characteristics was revealed and discussed.

## 2. Materials and Methods

### 2.1. Sensor Design and Fabrication

**Figure 1:**
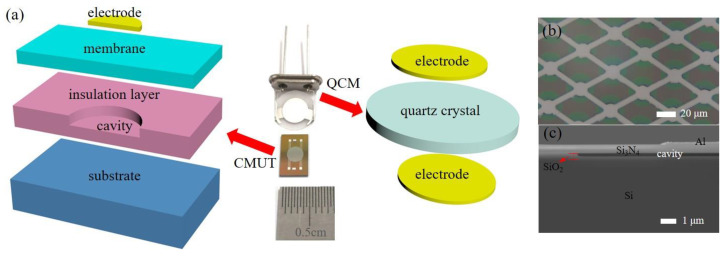
(**a**) Photograph of the CMUT and the QCM (middle); schematic of half the structure of a CMUT unit (left); schematic of the structure of a QCM (right); (**b**) microscopic image of the fabricated CMUT; (**c**) SEM image of the cross-section of the CMUT unit.

As presented in Figure 1a, a basic CMUT unit is composed of a top electrode, a rigid substrate that also serves as the bottom electrode, and a movable membrane that is isolated from the substrate by an insulation layer and a vacuum cavity. Generally, hundreds or thousands of such electrically connected units comprise a CMUT sensor. The structure of a QCM is much simpler. It consists of a quartz crystal sandwiched by the top and bottom electrodes.

Here, a 10 MHz AT-cut QCM with silver electrodes was derived from Wuhan Hitrusty Electronics Co., Ltd., (Wuhan, China) the photograph of which is shown in Figure 1a. The diameters of the quartz crystal and the electrodes were 8 mm and 4 mm, respectively. The electrode area was the active sensing area.

A 10 MHz circular CMUT was designed for comparison with the commercial QCM. The diameter of the CMUT was designed to be 3 mm, which ensured the fabrication yield and was also close to that of the QCM electrode for a relatively fair comparison. The resonance frequency of the CMUT is determined by [17]:

(1) $$f_{c}=0.47\frac{t_{c}}{a^{2}}\sqrt{\frac{E_{c}}{\rho_{c}\left(1-\nu_{c}^{2}\right)}}$$

where $a,\;t_{c},E_{c},\;\rho_{c},$ and $\nu_{c}$ are the radius, the thickness, the Young’s modulus, the density, and the Poisson’s ratio of the resonant membrane, respectively. Low-pressure chemical vapor deposition (LPCVD) silicon nitride (Si_3_N_4_) was selected as the membrane material due to the fabrication process. Consequently, the structural parameters of the membrane governed the resonance frequency of the device. As the resonance frequency was designed as 10 MHz, the thickness and radius of the membrane were determined accordingly to be 520 nm and 15 μm, respectively. A 100 nm thick electrode layer was deposited on the top of each membrane. The cavity depth was designed to be 180 nm to allow free vibration of the resonant membrane. The insulation layer was designed as 450 nm to avoid breakdown of the device. The CMUT was composed of 5380 units connected in parallel.

The CMUT was fabricated by the MEMS fabrication process based on the nitride-to-oxide wafer bonding technique [21]. To start with, a silicon substrate was grown with wet thermal oxide and defined with cavities in the silicon oxide (SiO_2_) layer. The substrate was then bonded in a vacuum with a silicon-topped wafer, which was deposited with an LPCVD Si_3_N_4_ layer on both sides. Subsequently, the resonant nitride membrane was released by removing the backside nitride layer and the silicon handling layer on the top wafer. After exposing the bottom electrodes, Cr/Al metal electrodes and contact pads were deposited and patterned. Figure 1b shows the microscopic image of a partial CMUT. It can be seen that the aluminum electrodes are sitting atop the circular membranes. The CMUT consisted of over five thousand electrically connected units. Figure 1c presents the scanning electron microscopic (SEM) image of the cross-section of a CMUT unit. The metal electrode, the Si_3_N_4_ membrane, the SiO_2_ insulation layer, and the silicon substrate can be identified in the image.

### 2.2. Preparation of PVA Film

The PVA powders with a viscosity of 54.0 mPa·s were derived from Sinopharm Chemical Reagent Co., Ltd., (Shanghai, China). Then, 10 mg of the PVA powder was dispersed in 19.99 g ultrapure water and stirred by a magnetic stirring bar at 90 °C for 3 h until the powders were completely dissolved. Afterward, the dispersion was left at room temperature for 12 h for cooling down and degassing. Then, 7 μL of the 0.05 wt% PVA dispersion was drop-casted on the surface of the CMUT and the QCM, respectively. Lastly, the sensing films were dried by baking at 60 °C for an hour.

### 2.3. Characterization

The surface morphology of the PVA layers on the CMUT and QCM sensors were examined by SEM (Apreo S HiVac, Thermo Fisher, Waltham, MA, USA). The electrical properties of the sensors were characterized by a vector network analyzer (VNA). As the CMUT requires a bias voltage to bring its resonating membrane close to the substrate for higher efficiency, it was biased at 35 V by a DC power supply (GPR-11H30D, GW Instek, Taipei, Taiwan, China) and connected to a streamline USB VNA (P5000A, Keysight, Santa Rosa, CA, USA) through a bias tee, while the QCM was directly connected to the VNA. The S_11_ parameters of the sensors were measured by the VNA and converted to electrical impedances and admittances.

### 2.4. Humidity Sensing Measurement

**Figure 2:**
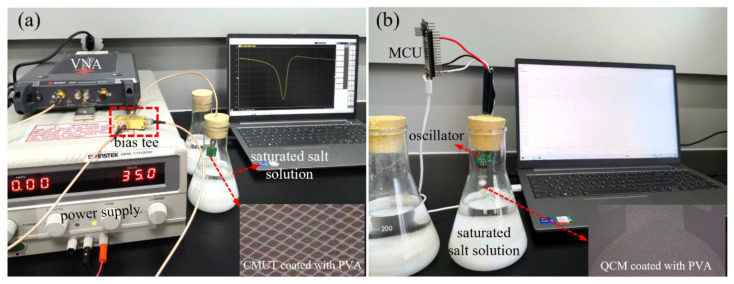
Experimental setups for the humidity sensing measurements of the CMUT (**a**) and QCM humidity sensors (**b**).

The humidity sensing measurements were conducted by monitoring the sensors’ frequency changes while alternating the RH environments at room temperature. RH levels of 11%, 23%, 33%, 43%, 58%, 75%, 84%, and 97% were generated by saturated salt solutions of LiCl, CH_3_COOK, MgCl_2_, K_2_CO_3_, NaBr, NaCl, KCl, and K_2_SO_4_, respectively [31]. Figure 2 shows the experimental setups for the measurements of the CMUT and QCM humidity sensors. The CMUT was biased at 35 V and its resonance was monitored by the VNA. Because the frequency shift of the QCM sensor was at the Hz level, the number of points to be swept by the VNA should be extremely large to achieve a satisfying frequency resolution, which will lead to a long sweeping time and bulky data process. Herein, the QCM was interfaced with an oscillating circuit and the resonance of the oscillator was tracked by an Arduino microcontroller unit (MCU).

## 3. Results and Discussion

### 3.1. Characterization

**Figure 3:**
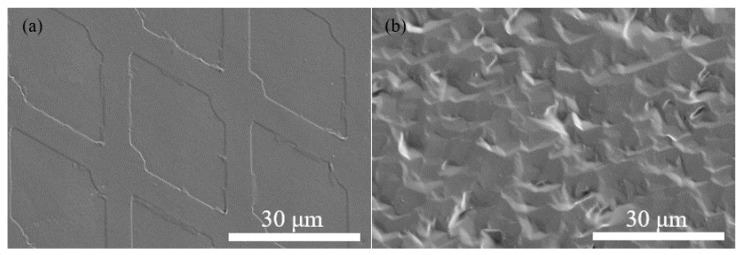
SEM images of the PVA films on the surfaces of the sensors: (**a**) CMUT; (**b**) QCM.

Figure 3a,b displays the SEM images of the PVA films on the surfaces of the CMUT and QCM sensors, respectively. Because of the MEMS fabrication process, the PVA film looks smooth on the surface of the CMUT. In comparison, the PVA layer on the QCM presents clear tortuosity due to the rough surface of the quartz crystal.

**Figure 4:**
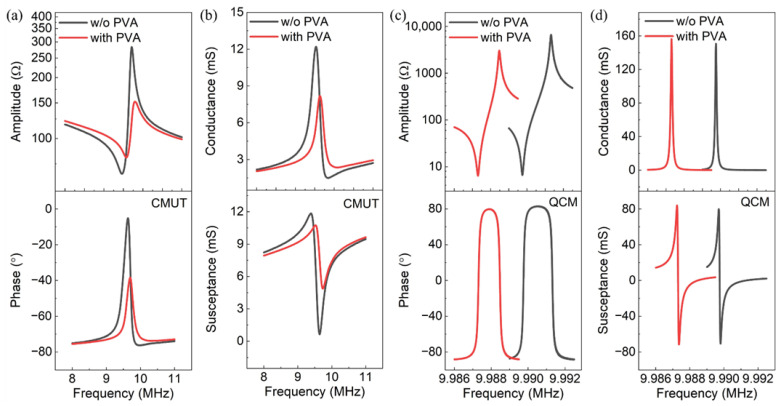
Electrical characterizations of the CMUT: (**a**) impedance and (**b**) admittance; electrical characterizations of the QCM: (**c**) impedance and (**d**) admittance.

Figure 4 presents the impedances and admittances of the CMUT and QCM before and after coating of the PVA film. It is shown in Figure 4a,c that the resonance frequencies of the bare CMUT and QCM were 9.714 MHz and 9.991 MHz, respectively, both of which were close to the designed 10 MHz. After functionalization by the PVA layer, while the resonance frequency of the QCM decreased by 2.85 kHz due to the loaded mass of the sensing material, the CMUT exhibited a frequency increment of 82 kHz. The increased resonance frequency of the CMUT can be explained by a multilayered thin plate model [32,33], the details of which are presented in Appendix A. During operation, the thin PVA layer vibrates together with the resonant membrane of the CMUT. Therefore, the two layers can be considered as a laminate structure. How the PVA film affects the resonance of the bare CMUT depends mainly on its material properties. The film with higher Young’s modulus and lower density will increase the resonance.

From Figure 4a,b, it is shown that the maximum amplitude and phase angle of the impedance and the quality factor of the CMUT were 283 Ω, −5.18°, and 39, respectively. After deposition of the PVA film, the maximum amplitude and phase angle of the impedance decreased by 46% and 33°, respectively, indicating a weakened resonance, while the quality factor slightly changed to 41. Similar results are observed for the QCM in Figure 4c,d. The maximum amplitude and phase angle of the impedance decreased by 54% and 3°, respectively, and its quality factor decreased from 97,939 to 87,608. The CMUT had a much lower quality factor than the QCM here.

**Figure 5:**
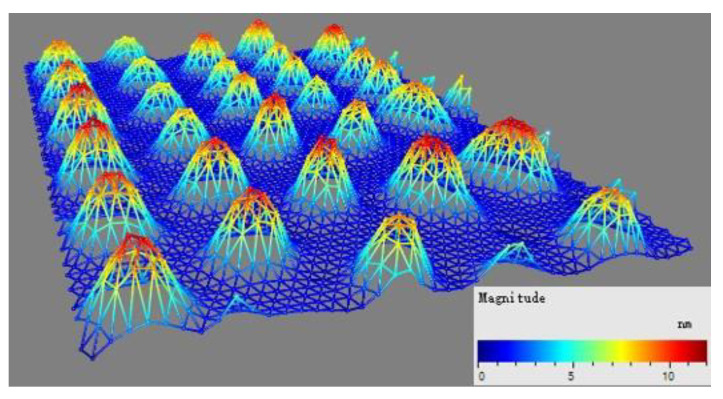
Laser vibrometer measurement of the CMUT: 2D scan of the membrane displacement under 1 Vpp sinusoidal signal at its resonance.

Generally, a high quality factor of 300–500 is achievable by the CMUT despite the massive parallelism [34], because only one side of its resonant membrane faces air damping while the other side is vacuum-sealed. The low quality factor here was likely caused by its fabrication process. Our equipment (Phantom II Reactive-ion Etching system, Trion Technology Inc., Clearwater, FL, USA) had unstable power during the reactive-ion etching (RIE) process, which led to the non-uniform etching of the cavities and further a relatively low quality factor. The hypothesis was verified by the laser vibrometer measurement (DD-300 displacement decoder, OFV-5000, Polytec Inc., Irvine, CA, USA). Figure 5 presents the 2D scan of the membrane displacement of the CMUT when it was biased at 35 V and excited by a 1 Vpp continuous sinusoidal signal. A strong vibration was observed for all scanned membranes, showing the good functionality of the device. Although the difference among individual membranes was small, it caused energy dissipation and decreased the quality factor of the sensor. The uniformity could be improved by optimizing the etching recipe or the fabrication process, such as replacing the RIE etching step with wet etching and redeposition of the oxide insulating layer.

### 3.2. Humidity Sensing Measurements

- Reproducibility, Sensitivity, and Linearity

**Figure 6:**
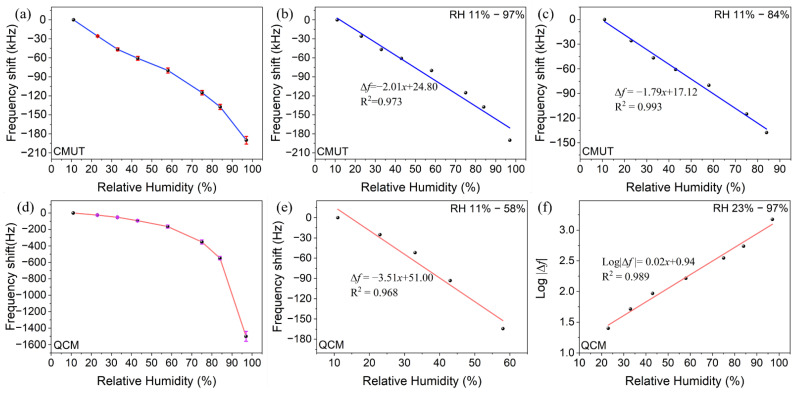
Sensitivity measurements of the CMUT sensor: (**a**) frequency shift as a function of RH from 11%RH to 97%RH. Error bars represent three replicate measurements; (**b**) correlation function curve between the frequency shift and RH from 11%RH to 97%RH; (**c**) correlation function curve between the frequency shift and RH from 11%RH to 84%RH. Sensitivity measurements of the QCM sensor: (**d**) frequency shift as a function of RH from 11%RH to 97%RH. Error bars represent three replicate measurements; (**e**) correlation function curve between the frequency shift and RH from 11%RH to 58%RH; (**f**) correlation function curve between the logarithmic frequency shift and RH from 23%RH to 97%RH.

Figure 6a,d shows the frequency responses of the CMUT and QCM sensors to various RH levels from 11%RH to 97%RH, respectively. The resonance frequency at 11%RH was used as the benchmark. Error bars in the figures represent the standard deviations of three separate measurements. Both sensors exhibited satisfying reproducibility with maximum deviations of ±3.8% and ±4.3% at 97%RH, respectively. 

It is known that both the CMUT and the QCM sensors rely on the detection of resonance shifts in response to the loaded mass Δm. The frequency shift of the CMUT is estimated by

(2) $$\Delta f_{c}=-\frac{f_{c}}{2A_{c}t_{c}\rho_{c}}\cdot \Delta m$$

where $A_{c}$ is the surface area of the resonant membrane. Assuming that the sensing layer can be approximately seen as part of the oscillating crystal, the frequency change in the QCM follows the Sauerbrey equation [11]:

(3) $$\Delta f_{q}=-\frac{2f_{q}^{2}}{A_{e}\sqrt{\mu_{q}\rho_{q}}}\cdot \Delta m$$

where $f_{q},\;A_{e},\;\mu_{q},$ and $\rho_{q}$ are the fundamental resonance frequency, the electrode surface area, the shear modulus, and the density of the QCM, respectively.

From Equations (2) and (3), the theoretical mass sensitivity of the CMUT was calculated to be 4.2 Hz/fg, which was six orders of magnitude higher than that of the QCM (1.15 Hz/ng). Within the RH level of 58%, however, it is observed from Figure 6 that the frequency shift in the CMUT sensor was only about three orders of magnitude higher than that of the QCM sensor. The discrepancy between the mass sensitivity and the RH sensitivity can be explained by the effective oscillation area and additive mass of the sensors. The CMUT units work in parallel, and the effective oscillation area of each unit is at the microscale, which provides a very small area for adsorption of water molecules and thus results in ultra-low mass loading. In comparison, the QCM device works as a single element with a large electrode area being the effective oscillation area. As a result, it has a considerably higher mass loading than the CMUT unit. At 23%RH, for instance, the frequency shifts in the two sensors were 25.8 kHz and 25.1 Hz, respectively, which can be translated to 6.2 pg and 21.8 ng of mass loading. Despite the much lower mass loading, the CMUT sensor still has the best the RH sensitivity due to its ultra-high mass sensitivity.

From Figure 6b,c, it can be seen that the CMUT sensor has good linearity, especially in the RH range of 11% to 84%. Its maximum frequency shift in the full RH range was 190 kHz at 97%RH. Accordingly, the loaded mass of water molecules was calculated to be 45.2 pg, which was negligible compared to the mass (1.2 ng) of the resonant membrane. The broad linearity of the response can be partially attributed to the relatively small mass loading. The RH sensitivity of the CMUT sensor was calculated to be 2.01 kHz/%RH in the RH range of 11% to 97%. In comparison, although the QCM sensor revealed an approximately linear response from 11%RH to 58%RH (Figure 6e), its response was exponential over the full RH range. Figure 6f presents the logarithmic linear fitting function of the response curve, the correlation coefficient of which was 0.989. The nonlinear behavior of the QCM humidity sensor can be explained by the Brunauer–Emmett–Teller (BET) adsorption model [35]. The exponential response curve of the sensor resembles the type III BET isotherm, indicating that the mass of adsorbed water molecules increases nonlinearly as the RH increases, which leads to a nonlinear frequency shift response.

- Dynamic response and hysteresis

**Figure 7:**
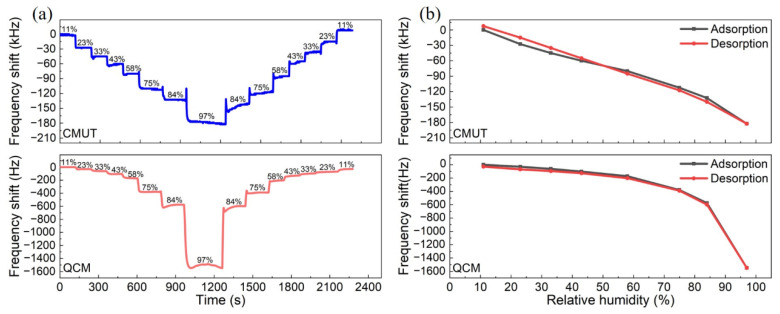
(**a**) Real-time frequency shift measurements of the sensors during the adsorption and desorption processes; (**b**) hysteretic curves of the sensors.

Figure 7a compares the real-time frequency responses of the sensors as the RH levels gradually changed from 11% to 97% and then back to 11%. The frequency shift of the CMUT sensor between adjacent RH levels can be easily identified due to its linear response, while it was hard for the QCM sensor at RH levels below 43%. Figure 7b illustrates the hysteretic curves of the sensors. The adsorption and desorption curves overlapped well for both sensors, indicating low hysteresis.

- Response/Recovery time and Repeatability

**Figure 8:**
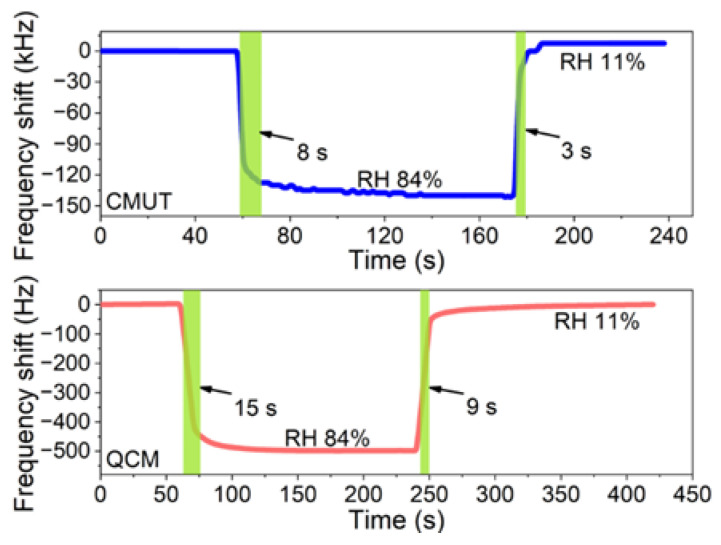
Dynamic responses of the CMUT and QCM sensors as the RH level alternates between 11% and 84%.

The dynamic frequency responses of the CMUT and QCM sensors were evaluated as the RH switched between 11% and 84%. Figure 8 highlights the 90% response and recovery zones. The response/recovery times of the CMUT and QCM sensors were 8 s/3 s and 15 s/9 s, respectively. The faster response and recovery speed of the CMUT sensor was the benefit of its microstructure. As the microscale resonant membrane of the CMUT was ultra-lightweight, the air damping it withstood was considered significant, which helped to stabilize the resonance frequency faster during the change in RH.

**Figure 9:**
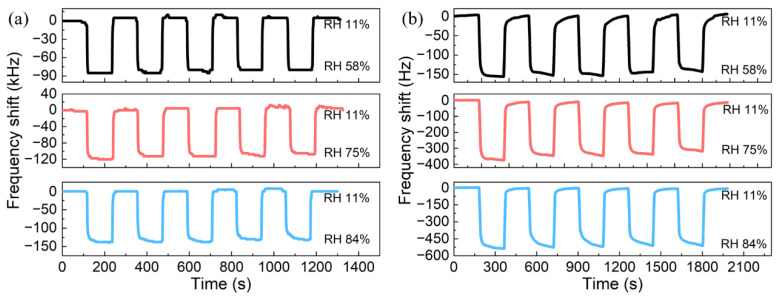
Repeatability of the sensors as the RH level switches from 11% to 58%, 75%, and 84%: (**a**) CMUT; (**b**) QCM.

The repeatability of the sensors was examined by switching the RH from 11% to 58%, 75%, and 84% for five cycles. As shown in Figure 9, both the CMUT and QCM sensors exhibited decent repeatability in all cases. Meanwhile, the response/recovery was slower for both sensors at higher RH levels due to the longer penetration process of water molecules into the sensing film. It was also observed that the dynamic response/recovery of the CMUT sensor was faster than the QCM sensor despite the change in alternating RH levels.

- Temperature dependence and long-term stability

**Figure 10:**
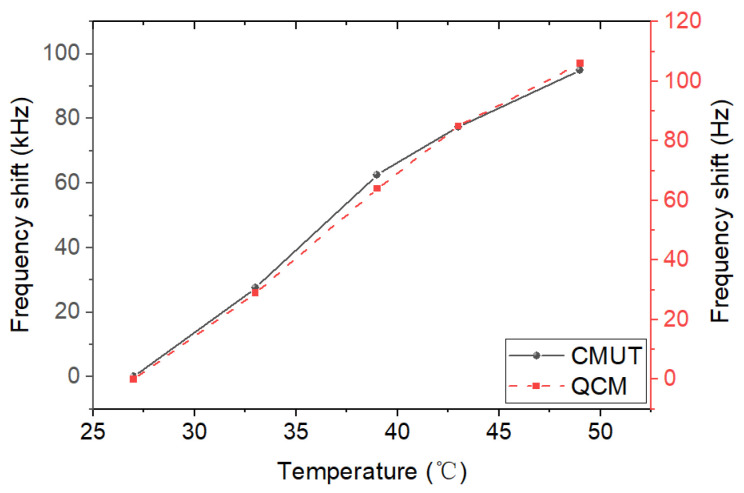
Frequency shifts of the CMUT and QCM sensors as a function of temperature.

It is well known that the properties of semiconductors and quartz are temperature-dependent. Therefore, the temperature dependence of the CMUT and QCM sensors was studied. Figure 10 shows the frequency shifts in the two sensors as the temperature rises from 27 ℃ to 49 ℃. The resonance frequencies at 27 ℃ were used as the benchmark. It was observed that the resonances of both sensors exhibited the same increasing trend towards temperature and the frequency shifts increased almost in a linear fashion. Temperature compensation for the sensors may be needed to ensure the accuracy of the humidity sensing measurements when temperature is not stabilized. 

**Figure 11:**
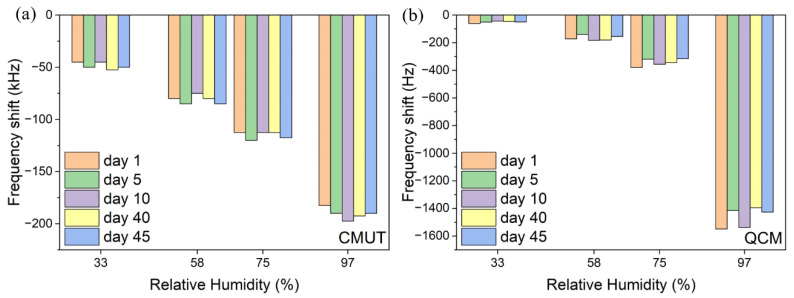
Long-term stability performance of the sensors: (**a**) CMUT; (**b**) QCM.

The long-term stability of the sensors was investigated by measuring their frequency shifts in different RH environments a few times over 45 days. Figure 11 displays the long-term stability performance of the CMUT and QCM sensors. The frequency shifts of both sensors were in good agreement at the same RH level on different days, indicating good long-term stability. The largest deviations over time were at 97%RH. Therefore, it is preferred to keep the sensors in lower RH environments.

Table 1 summarizes the humidity sensing performance of the proposed sensors in this work and some of the resonant devices reported in recent years. The comparison demonstrates the outstanding all-around RH sensing performance of the CMUT-based humidity sensor in this work.

## 4. Conclusions

This work reports a high-performance CMUT-based humidity sensor using PVA as the sensing film. In addition, to the best of our knowledge, this work provides the first side-by-side comparative study of the CMUT and QCM sensor platforms. The two types of gravimetric humidity sensors were designed and fabricated. Their electrical properties were characterized by a VNA and the humidity sensing performance was examined over a wide RH range from 11% to 97%. Although the working principles of both sensors were based on the mass-loading effect, the QCM worked as a single element while the CMUT involved massive parallelism, which led to a huge difference between their effective oscillation areas of four orders of magnitude. Consequently, a considerable discrepancy between their RH sensitivity and theoretical mass sensitivity was observed. Despite the small effective oscillation area of each unit, the CMUT humidity sensor exhibited a high RH sensitivity of 2.01 kHz/%RH, which was about three orders of magnitude higher than that of the QCM sensor. In addition to the high sensitivity, the unique microstructure has also provided the CMUT humidity sensor with excellent all-around performance, including a short response/recovery time of 8 s/3 s, good reproducibility and linearity, and low hysteresis. The experimental results demonstrated the superiority of the CMUT sensor platform over the QCM counterpart. The major drawback of the CMUT sensor was the need for a DC bias voltage to pre-displace the resonant membrane. The bias voltage was 35 V in this work. It could be improved by optimizing the structural parameters of the device. For instance, by minimizing the cavity depth, reducing the bias voltage to 6-8 V is possible, which would be favorable for portable humidity sensing applications.

## Figures and Tables

**Table 1:** Comparison between the humidity sensors in this work and reported gravimetric humidity sensors.

Ref.	Sensor	Resonance Frequency (MHz)	Sensing Material	Sensitivity (Hz/%RH) /RH Range (%)	Response /Recovery (s)	Linearity
This work	CMUT QCM	10 10	PVA PVA	2.01 k (11–97) 3.51 (11–58)	8/3 15/9	Good Bad
[36]	CMUT	10	CNCs	0.9 (11–53) 2 (53–94)	7/2	Bad
[20]	CMUT	50	mesoporous silica guanidine polymer	1.2 k (0–20) 2.6 k (0–20)	88/105 53/53	Good Good
[37]	QCM	10	CNCs	55 (11–54) 275 (54–97)	60/15	Bad
[38]	QCM	10	ZIF-CoNi	6.10 (11–97)	3/3	Medium
[39]	QCM	8	SnO2	23.2 (11–97)	10/3	Bad
[40]	QCM	10	BiOCl	7.3 (11–97)	5.2/4.5	Bad

## Data Availability

The data presented in this study are available on request from the corresponding author.

## Appendix A

The thin PVA layer and the membrane of the CMUT can be seen as a laminate thin film, the resonance frequency of which is estimated by [32,33]:

$$f=\frac{1.63}{a^{2}}\sqrt{\frac{C-B^{2}/A}{\rho_{c}t_{c}+\rho_{p}t_{p}}},$$

where:

$$A=\frac{E_{c}t_{c}}{1-\nu_{c}^{2}}+\frac{E_{p}t_{p}}{1-\nu_{p}^{2}},$$

$$B=\frac{E_{c}t_{c}^{2}}{2\left(1-\nu_{c}^{2}\right)}+\frac{E_{p}\left[{\left(t_{c}+t_{p}\right)}^{2}-t_{c}^{2}\right]}{2\left(1-\nu_{p}^{2}\right)},$$

$$C=\frac{E_{c}t_{c}^{3}}{3\left(1-\nu_{c}^{2}\right)}+\frac{E_{p}\left[{\left(t_{c}+t_{p}\right)}^{3}-t_{c}^{3}\right]}{3\left(1-\nu_{p}^{2}\right)},$$

where $\;\rho_{p},\;t_{p},\;E_{p},$ and $\nu_{p}$ are the density, the thickness, the Young’s modulus, and Poisson’s ratio of the PVA film, respectively.
